# Strangulated Hernia

**Published:** 1828-02

**Authors:** 


### STRANGULATED HERNIA.

We subjoin two cases of this kind, which bear upon an interesting and important practical question.

Case I.—William Fox, ætat. twenty-two, a hale young countryman, was brought to St. George's Hospital, at twelve m., December 5th, from Stanmore. He states that he has been subject to rupture from childhood, and that, till within the last twelvemonths, he wore a truss, although there always remained some swelling at the part on which the instrument was applied. For the last year he has laid aside the truss altogether, and felt no inconvenience; nor did any additional protrusion occur until five o'clock last night, when, after wrestling, he found a swelling in the groin. He vomited soon afterwards. A surgeon who saw him bled him freely, and tried the taxis, which however failed. He passed a bad night, and the taxis was again ineffectually employed this morning.

On admission, there was seen a large scrotal hernia on the left side. The tumor was very tense, and tender upon pressure; the integument not discoloured, and the testicle could be felt below and rather behind the tumor, on which it was moveable: in this respect differing from congenital hernia. No pain on pressure of the abdomen; pulse ninety, not very hard; tongue white; no anxiety of countenance. The bowels had not been opened since the 3d, and he vomited the drink given him. He was placed in the warm bath for nearly an hour, and, on being removed to bed, was bled to syncope. The taxis was then employed, but without effect.

Five P.M.—Cold by means of the sulphuric æther, and bags of pounded ice, has been applied for the last two or three hours, with occasional attempts at reduction. These failing, he was again bled to syncope, and the taxis had recourse to; but no impression being made upon the tumor, Mr. ROSE proceeded to the operation.

On cutting into the sac, much bloody serum escaped, and a layer of omentum was exposed, enveloping a large fold of small intestine. The omentum was comparatively but little altered; whilst the intestine, though still glossy and firm, was very dark and discoloured. The stricture, which was at the outer ring, and exceedingly tense, was divided, and the gut carefully returned, when it was discovered that a quantity of omentum still remained in the sac, to which it had contracted firm, and evidently old, adhesions. This it was not deemed prudent to return: it was accordingly disturbed as little as possible, and the integuments brought together by five or six sutures, with adhesive straps and compress.

**Small doses of Sulphate of Magnesia, in peppermint water, every two hours.**

He passed an excellent night, and had two or three stools; but, on the evening of the next day, he complained of a good deal of pain in the tumor, increased on pressure, and stretching from that up to the umbilicus. Pulse 100; skin hot and dry; great thirst.

**V.S. ad ℥xx.—H. Sal. cum Mag. Sulph. sextis horis.**

These means, with the application of twenty-five leeches, and fomentations to the abdomen, greatly relieved the pain there, but a good deal still remained in the tumor.

On the 8th, the pulse was 100, full and bounding; tongue brownish in the centre; nausea; occasional chilliness. The straps were removed, and the wound found to have in great measure united, but a degree of fulness was observed at its upper part.

**V.S. ad ℥xij.**

9th.—He fell into a perspiration after the bleeding, and passed a good night. Still looks anxious; pulse has more jerk; nausea; headache; thirst. There is a distinct blush with a little œdema about the wound, and at the outer and upper part of the tumor there is evident fluctuation. At this part a puncture was made, giving vent to an ounce or more of darkish pus, mixed with bubbles of air; not, however, having the odour of sulphuretted hydrogen.

**H. Sal. Effervesc. cum Succ. Limon. recent p. r. n.—Cat. Lini.**

In the evening, a small artery, which had been divided by the lancet, threw out nearly a pint of blood into the poultice. The bleeding was stopped after some trouble, by compresses of lint and rags dipped in cold water. From this period all the bad symptoms disappeared.

Case II.—Joseph Haplin, æt. forty-two, admitted twelve P.M. December 15th, under the care of Mr. Brodie, with inguinal hernia in the left side. The tumor was tense, oval, about the diameter of an orange, and there was little pain in it on pressure. There was great tenderness, however, of the abdomen; constant vomiting; hiccough; anxious countenance; skin pale and cold; pulse small and weak.

At five A.M. of the day of his admission, whilst going his rounds as a watchman, he was attacked with griping of the bowels, to which he was subject. He returned home and went to bed, but the pains increased, and at ten A.M. he began to vomit. The tumor was at this time rather larger than usual, and as the day advanced it became painful, and perceptibly increased in size.

The warm bath, taxis, &c. were employed by the house surgeon, but to no effect. At three A.M. Mr. Brodie arrived, and determined on an immediate operation. A knuckle of intestine, much redder than natural, was found at the upper part of the sac, and a portion of omentum, not thickened or otherwise diseased, extensively adherent to it below the stricture. The stricture, which was at the neck of the sac, was divided, and the gut returned; but the adherent omentum was left. The patient did well.

*Observations.*—It will be observed that in these cases the omentum was left unreduced. In both it was extensively adherent, and in neither was it disorganized. We fear that we should far exceed the space allotted us, were we to enter at all into what authors have advanced upon the treatment of strangulated omentum. Sir A. Cooper, in his very excellent observations upon Hernia, published in the third volume of his "Lectures," remarks that, if the omentum be very large, if it be in a state of mortification, or if it be "hardened, and have a scirrhous feel," it should be removed wholly or in part; the divided vessels secured by fine ligatures, and these, one end being cut off, should be left hanging from the wound. If, however, says Sir Astley, omentum alone adheres to the sac, it may be freely separated and returned. Mr. Brodie, whose experience cannot have been inconsiderable, comes to a different conclusion. In his clinical lecture upon Joseph Haplin's case, he observed, that whatever difficulty obtained in reducing the omentum, either from its being adherent, mortification, or from its being converted into a large fatty mass, the old practice used to be to cut it off, or apply a ligature upon it. With regard to the ligature, authors are almost unanimous in disapproving of it: in fact, as Sir A.

Cooper shrewdly remarks, it is renewing with increased severity what the operation was intended to relieve—the stricture on the part.

Mr. Brodie is inclined to think that the excision of the omentum is not so devoid of difficulty and danger as some would seem to infer. If no vessels are tied, the hemorrhage may be very considerable: indeed, Mr. HEY has published two cases in which the patients nearly died of it. If, again, the arteries are tied, the ligatures cut off close, and the omentum returned, we are not sure that bleeding may not take place within the abdomen, from vessels which did not bleed while the omentum lay exposed in the sac, and which therefore were not secured: and, besides, what is to become of the ligatures? Mr. EARLE related to Mr. Brodie a case of this kind, in which they were discharged through an abscess which presented itself at or near the umbilicus. The patient recovered; but, nevertheless, an abscess of the belly, which bursts at the umbilicus, cannot be supposed to be always free from danger. If, instead of the ligatures being cut close, one end of each of them be left hanging out at the neck of the hernial sac, the omentum must, of course, remain drawn down to the abdominal ring; and in what respect is the patient better off than if a portion of it had been left in the sac itself? There is also some danger in this case of inflammation of the cut end of the omentum; and Mr. Brodie mentioned one case in which an abscess formed in the omentum, at this part, immediately within the internal abdominal ring, and the patient died in consequence.

He, however, stated, that he did not mean to condemn the excision of the omentum in all cases, but merely to express that it ought not to be done without a very sufficient reason; and that in cases where there is not a very large quantity of omentum in the hernia, but where it is externally adherent to the surface of the sac, it is safer to leave it where it is found, than to cut off a portion of it, or to dissect through the adhesions. The success of this practice in the two preceding cases, is certainly in its favor. The patients are left as well off as they were before the strangulation took place; and probably better, inasmuch as it is most likely that the omentum, after the operation, must have contracted adhesions to the neck of the hernial sac, making them less liable to a descent of the intestine. (*Condensed from the Medical Gazette.*)

